# Experimental Investigation on Dry Routing of CFRP Composite: Temperature, Forces, Tool Wear, and Fine Dust Emission

**DOI:** 10.3390/ma14195697

**Published:** 2021-09-30

**Authors:** Tarek Elgnemi, Victor Songmene, Jules Kouam, Martin B.G. Jun, Agnes Marie Samuel

**Affiliations:** 1 Department of Mechanical Engineering, École de Technologie Supérieure (ÉTS), Montreal, QC H3C-1K3, Canada; tarek-shaban-mohamed.elgnemi.1@ens.etsmtl.ca (T.E.); jules.kouam@etsmtl.ca (J.K.); agnesmsamuel@gmail.com (A.M.S.); 2 Department of Mechanical Engineering, Purdue University, West Lafayette, IN 47907, USA; mbgjun@purdue.edu

**Keywords:** CFRP, machining, temperature, cutting forces, dust emission, tool wear

## Abstract

This article presents the influence of machining conditions on typical process performance indicators, namely cutting force, specific cutting energy, cutting temperature, tool wear, and fine dust emission during dry milling of CFRPs. The main goal is to determine the machining process window for obtaining quality parts with acceptable tool performance and limited dust emission. For achieving this, the cutting temperature was examined using analytical and empirical models, and systematic cutting experiments were conducted to assess the reliability of the theoretical predictions. A full factorial design was used for the experimental design. The experiments were conducted on a CNC milling machine with cutting speeds of 10,000, 15,000, and 20,000 rpm and feed rates of 2, 4, and 6 µm/tooth. Based on the results, it was ascertained that spindle speed significantly affects the cutting temperature and fine particle emission while cutting force, specific cutting energy, and tool wear are influenced by the feed rate. The optimal conditions for cutting force and tool wear were observed at a cutting speed of 10,000 rpm. The cutting temperature did not exceed the glass transition temperature for the cutting speeds tested and feed rates used. The fine particles emitted ranged from 0.5 to 10 µm aerodynamic diameters with a maximum concentration of 2776.6 particles for those of 0.5 µm diameters. Finally, results of the experimental optimization are presented, and the model is validated. The results obtained may be used to better understand specific phenomena associated with the milling of CFRPs and provide the means to select effective milling parameters to improve the technology and economics of the process.

## 1. Introduction

The use of a carbon fiber reinforced polymer (CFRP) has considerably increased in the last few years. The aerospace and automotive industries especially are concerned about these materials, due to the fact that (i) CFRPs are relatively easy to manufacture using several automated lamination techniques; (ii) they feature excellent unique mechanical properties as well as good chemical and dimensional stability; and (iii) their corrosion and heat resistance are also outstanding [1,2,3]. The main attraction of these materials is the low density compared to the traditional engineering materials such as steel or aluminum [4]. These characteristics allow for a reduction in costs [5,6], which is an important requirement in any kind of industry. However, due to the multiphase and inhomogeneous nature of the material, various types of damage, e.g., fiber breakage and pullout, fuzzing, delamination, resin degradation, etc., are easily induced [7,8]. In addition, the highly abrasive nature of the carbon fibers and the low thermal conductivity of the resin matrix lead to rapid tool wear; the laminated structure of the CFRP facilitates delamination as plies are subject to separation by cutting forces during machining [9,10].

Despite the fact that CFRPs are mostly produced near net shape, machining is often required in order to bring the component into dimensional requirements and prepare it for assembly. Milling operation remains essential and necessary to achieve the required geometry, tolerance level, and edge quality in CFRPs, which are important for assembly [11,12]. Significant progress has been made to understand the effects of fiber materials and matrix types [13,14], fiber volume fraction and orientations, [15], tool materials and geometries [16], and machining parameters [17,18]. Sheikh-Ahmad [19], for instance, demonstrated numerous studies on the machining of a CFRP. It was shown that the machining quality or machinability of CFRP materials relies on factors such as the fiber volume fraction, fiber orientation, cutting parameters, and tool geometry. Davim and Reis [12] found that both cutting speed and feed rate have a statistical and physical significance in the milling of a CFRP. In a further study of the milling of a CFRP, Azmi et al. [20] observed that the feed rate has the most dominant effect on surface roughness and machining force. Therefore, machining has to be conducted with great care to obtain high quality.

CFRPs are normally machined under dry conditions, i.e., without using a coolant [21]. This is due to the fact that the moisture can degrade the mechanical properties of composites, as seen by microcracking of the polymer matrix and chemical reaction of the polymer [22]. On the other hand, dry machining generates overheating. As a result, thermal damage to composites [23] and tool life occur [24].

In addition to the above-mentioned cutting mechanics, the cutting temperature has long been recognized as an important factor influencing the surface quality of milled materials and the tool life. If the cutting temperature is higher than the thermosetting matrix resin’s glass transition temperature, the resin will degrade within the machined surface or surface layer. This critical temperature is around 180 °C for a typical epoxy-based CFRP material. Degradation of the resin generates delamination and weakens the material, resulting in significant flaws [25]. However, it is reported that cutting temperature is significantly affected by the cutting speed, depth of cut, tool/workpiece material, feed rate, and fiber orientations [21].

In order to investigate the CFRP cutting temperature, various techniques have been used to measure temperature while milling CFRP, such as a thermal camera [26], a K-type thermocouple, and a tool-work thermocouple [27]. Chen [25] installed a thermocouple in the flank surface of a drill and performed the temperature measurement of the flank surface using the thermocouple technique. The results showed that when the cutting speed was increased from 40 to 200 m/min at a fixed feed rate of 0.05 mm/rev, the average flank surface temperature increased from 120 to 300 °C. When the feed rate was increased from 0.05 to 0.4 mm/rev at a fixed cutting speed of 21.5 m/min, the average flank surface temperature decreased from 120 to 70 °C. To detect the temperature of the milling operation, Kerrigan et al. [28] inserted a thermocouple in the cutting tool and created a wireless telemetric system within the rotary cutting tool. They concluded that in high-speed milling, responsiveness is insufficient to quantify dynamic change. Weinert and Kempmann [23] reported that the cutting temperature reaches its maximum when there is friction between the tool tip and the workpiece. They added that in this case, fibers are not cut but pressed out by the tool that generates its wear. In another study, Yashiro et al. [27] measured the cutting temperature during machining of a CFRP composite laminate and the temperature distribution through the laminate thickness during machining. They used the following three measurement methods: one with an infrared camera, a second one with a tool–workpiece thermocouple, and a third with thermocouples embedded between the layers of the composite. Their analysis indicated that the temperature at the tool–workpiece contact point reached 180 °C (Tg) at a cutting speed of 25 m/min and subsequently climbed to 300 °C at a cutting speed of 50 m/min. When the cutting speed was increased further, the cutting temperature tended to stabilize and remain consistent. Even at high cutting rates (300 m/min), the cutting temperature in the workpiece material was comparatively low (104 °C) compared to the tool–workpiece contact point. In recent research, Liu et al. [29] investigated the workpiece temperature variation in helical milling of CFRP. They concluded that the workpiece temperature increases with an increasing spindle speed and axial cutting depth. They also reported that the axial cutting depth has more influence on the temperature variation of the workpiece than the spindle speed, while the influence of feed per tooth is less than the other factors.

To date, measuring cutting tool temperature is a significant challenge in milling operations due to tool rotation and complicated set-up. Therefore, numerical and analytical models have been proposed, widely considered, and used until now. Lin et al. [30] developed a model to predict the cutting temperature of the workpiece during the end milling process while considering the flank face wear. The model’s accuracy exceeds that of an analytical model, and its prediction efficiency exceeds that of the finite element approach, which can be used to optimize cutting parameters. Wang et al. [31] investigated the relationship between the cutting parameters, cutting temperature, and cutting force in CFRP milling using response surface methodology (RSM), and indicated that the cutting speed has a significant impact on the cutting temperature. Sheng et al. [32] suggested a modelling coupling equation of cutting parameters based on the ideal cutting temperature to optimize tool life.

In addition to all the exposed issues of composite machining, dry machining of CFRP composites leads to the generation of significant amounts of airborne dust particles. These particles can be inhaled and can even penetrate the skin or the eye, which is a direct cause of a health hazard. Moreover, the dust generated from machining CFRP is harmful to the machine tool as well [33]. Carbon fibers are electrically conductive, and due to the small size of the dust particles and the fibers, and their ability to become airborne, these particles will likely penetrate tight spaces between machine components and into the machine control box [34]. Numerous works have been conducted to identify the main factors responsible for the generation of dust particles in order to minimize the emission of these dangerous particles during the machining of CFRP [35,36,37]. Boatman et al. [38] conducted a study to identify the influence of the nature of composite materials on the size and the number of harmful particles during the trimming process. CFRP and GFRP materials were machined in this work. They found that harmful particles resulting from GFRP composites have a higher number and longer lengths than those resulting from CFRP composites.

On top of that, the number of harmful particles is almost equally distributed along with particle size (0.5 to 8 µm). However, when machining a CFRP composite, the majority of the harmful particles lie below 2 µm (almost 85%). It may be noticed that these studies dealing with dust particles generated throughout machining composite materials do not provide all the pertinent information on the influence of machining parameters on the emission of harmful particles. Additionally, no information has been provided on the tool geometry used. In another study, Haddad et al. [39] investigated the influence of tool geometry and cutting conditions on surface defects and the dust generated during CFRP milling. It was observed that an increase in the number of particles occurred with an increasing cutting speed. The feed rate appeared to have less of an effect at a constant cutting speed on the number of particles generated. On the other hand, a low cutting speed and high feed rate supported the apparition of mechanical damages that are responsible for the poor surface quality [19].

J. L. Miller [40] carried out a variety of tests on the machining of composite materials and the different particulate sizes of the machined dust. The tests were conducted using uni-directional and multi-directional laminates. He observed that the aerodynamic diameter of particles obtained by machining the uni-directional composites was about 0.15 μm. He also questioned the credibility of the measurement of the percentage of particles that are harmful to alveoli by Haddad et al. [39]. Regarding the influence of the tool geometry, however, it has been observed that the amount or number of harmful particles measured, when using four flutes end mills, is 150 and 120% superior to those generated when trimming is conducted with coated and uncoated burr tools [37]. These results have been attributed to the fact that, once the burr tools are used, the temperature of machining is superior to the one generated when machining is conducted with four flutes end mills, and this increases the adherence of the carbon and matrix dust in between the tool grooves of the burr tools [39]. It should be mentioned that these burr tools have been initially designed by the manufacturer of tools to minimize the cutting forces and also the delamination when trimming or milling CFRP, and not for the decrease in the amount or number of harmful particles. Thus, in our point of view, we can say that an optimal design of the cutting tool groove may reduce the cutting forces and also the number of harmful particles.

Summarizing the previous work available on the machining of composites, numerous studies have been conducted on the relationship between machining quality and cutting force and tool wear. However, few investigations have focused on the interaction effect of cutting force, cutting temperature, and dust emission in CFRP milling simultaneously.

The present work is primarily motivated by the need to focus on the interaction effect of cutting parameters (cutting speed and feed rate) on the following machining process performance indicators: cutting force, specific cutting energy, cutting temperature, tool wear, and fine dust emission (in terms of number of particles) during dry milling of CFRP. A complete experimental design has been developed with two factors (feed speed and cutting speed), each with three levels in order to obtain nine combinations, for milling of a multidirectional CFRP laminate. A chip breaker router type of end mill was considered to investigate the effect of machining parameters on cutting force, tool wear, cutting temperatures, and the dust generated. Down milling was deliberately chosen, being the preferred method for finishing operations, while machining temperatures were acquired using K-type thermocouples. Additionally, an analytical model was proposed to predict the temperature and then, experimental tests were used to verify the results obtained using the model. In this study, the relationships among milling temperature, milling force, fine dust emission, and cutting parameters are analyzed using response surface methodology (RSM), and the corresponding mathematical models are established to optimize the cutting parameters. Finally, the effect of cutting parameters on the dust generated while machining composite parts has also been investigated.

Section 2 proposes an analytical temperature field modeling method. Section 3 describes the material and outlines the experimental techniques for the testing of milling. Section 4 covers the analysis and experimental validation of the proposed model, and results. Conclusions are presented in Section 5.

## 2. Analytical Modeling of the Temperature and Specific Energy

The machining process performance is influenced by a large number of factors including the temperature in the cutting zone, the chip formation, the cutting forces, and the tool wear. All these factors are affected by the machining parameters, the cutting tool geometry and material, and the machining conditions. For the machining of the CRFP, the temperature is of great importance, as a high temperature could lead to matrix softening, delamination, and consequently, deterioration of the CFRP. The cutting temperature, the cutting energy, and the cutting and shearing forces also influence the dust emission during machining. Some of this information can be determined experimentally, while other information must be estimated indirectly.

An estimation of such critical factors could help selecting machining parameters that guarantee the production of quality parts, good productivity, and an acceptable level of dust emission.

Some analytical approaches were developed for the prediction of the cutting temperature. For example, the input parameters of the temperature model can be calculated according to the Nathan Cook model using experimental data to predict the cutting temperature [41]. In this model, we assumed that the ambient temperature is Ta = 22 °C. The cutting temperature (*T*) can be calculated as follows:

(1) $$T=0.4\frac{U}{\rho \cdot C}\cdot {(\;\frac{V\cdot f_{z}}{K})}^{0.333}$$

(2) Ttotal=Ta+T

where *U* is the specific energy of the material, *ρ* is the density the material, *C* is the volumetric specific heat of the material (j/(mm^3^·°C)), *V* is the cutting speed, *f_z_* is the feed per tooth, and *K* is the material diffusivity.

The diffusivity *K* may be determined from the specific heat Cρ, the density *ρ*, and the thermal conductivity α as follows [42,43]:

(3) $$K=\frac{\alpha }{\rho \;\;C\rho }$$

It is known that the specific cutting energy is the amount of energy used in removing a unit volume of the workpiece material per unit time during machining.

For conventional alloys, such as aluminum alloys and steels, the specific cutting energy is well documented, but it is not the case for composites or for a CFRP. Meanwhile, the other input parameters of the cutting temperature model can be calculated based on the specific energy *U*, which can be calculated from the cutting force (*Fc*), the cutting speed (*V*), and the metal removal rate (MRR) [41].

(4) $$U=\frac{Fc\cdot V}{MRR}=\frac{Fc\;V}{Ac\;V}=\frac{Fc\;}{Ac}$$

where *Ac* is the undeformed chip area that is given by the following [44]:

(5) Ac=ac+at

where *at* is the thickness of the unidirectional laminate and *ac* is the chip thickness, which varies continuously with the engagement angle ∅ of the tool, and is expressed as follows [45]:

(6) $$ac=f_{z}\;sin\emptyset$$

where $f_{z}$ is the feed per tooth.

The cutting forces *Fc* can be measured or estimated from the shear angle (∅) as follows [46]:

(7) Fc=−Fx sin ∅+Fy cos ∅

where *Fx* and *Fy* are forces to be measured using a table dynamometer.

Since the undeformed thickness of the chip is almost the same as the depth of cut used, (given the brittle nature of the CFRP [19,47]), the shear angle ∅ can be estimated through the tool rake angle (*α*) by the following [48]:

(8) $$\emptyset \approx \;tan^{-1}\;\left(\frac{cos\alpha }{1-sin\alpha }\right)$$

For this study, the cutting forces were measured, and the temperature was estimated then validated using experimental machining data. The fine dust emission was sampled during machining and the tool wear was estimated after a given number of machining passes.

## 3. Experimental Setup

**Figure 1:**
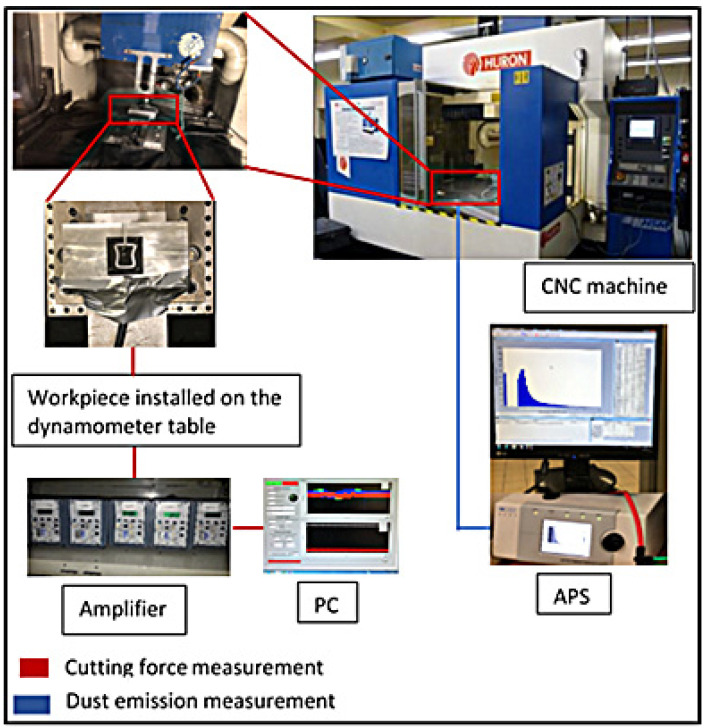
Photographs showing setup for machining CFRP and measurement system, and shape and geometrical dimensions of workpiece used in this study.

Down milling experiments were carried out using a HURON-K2X10 3-axis CNC machine tool with maximum spindle speed (N), 28,000 rpm; power (P), 50 kW; and torque (T), 50 Nm. A dynamometer (type Kistler 9255B) was clamped on the machine table and connected to the charge amplifiers (Kistler 5010) (Kistler Materials 2020, 13, 1181 5 of 22 Instrument Corporation, New York, NY, USA) that generated output signals, which were transmitted to a data translation card (type DT 9836, Data Translation Inc., Marlborough, MA, USA), linked to a personal computer. All signals, monitored independently, were digitized and recorded using LabView software program in order to analyze force measurements. An Aerodynamic Particle Sizer (APS, model 3321, TSI Inc., Shoreview, MN, USA) capable of measuring the aerodynamic size of particles from 0.5 to 20 microns. The dust samples were sucked by a pump (1.5 L/min) through a 10-millimeter suction tube, with the end of the tube placed near the machining area. The suction tube was connected to the dust measurement system, which consisted of an aerodynamic particle sizer (APS) spectrometer. The collected data were then analyzed using the TSI’s Aerosol Instrument Manager software. The experimental scheme is illustrated in Figure 1. The dust sampling was performed during the machining process (doors closed). The APS record signal was initiated 3 s before the cutting process started and was completed, after the end of the testing, when the dust measurement was near to zero.

### 3.1. CFRP Material and Tool Details

**Figure 2:**
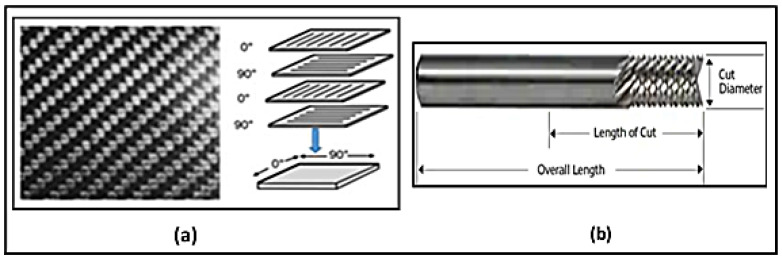
(**a**) Composite lay-up, (**b**) chip breaker routed geometry.

Multi-layer CFRP sheets of 1.56 mm (1/16 in) thickness were milled in each experiment. Each CFRP sheet consists of four unidirectional tapes of equal thickness that are laid up in (0F/90/0F/90/0F/90/0F/90) configuration. Figure 2a demonstrates the fabric prepreg orientation scheme. The workpiece materials were cut into small sheets of 38 mm length × 38 mm width. The total cutting length tested during experiments was Lc = 105 mm routing. Normally, the use of cutting fluid is not allowed in the secondary process of aircraft CFRP part machining; therefore, dry cutting was employed in this part of the study. Table 1 shows the mechanical and physical properties of the workpiece as obtained from the CFRP supplier (McMaster-Carr, Elmhurst, IL, USA).

A 3.175-millimeter diameter router end mill (ET-6-1250-F manufactured by Performance Micro Tool, Janesville, WI, USA) with chip breaker and with reverse V-shaped ends was used for milling operations, see Figure 2b. The edges are serrated to break up the chips into smaller pieces and for fast removal of material during roughing. This router is designed specially to produce profiling in composite materials. Fishtail ends with V-shaped ends are suited to produce a flat surface at the bottom and improve chip removal from the workpiece. The dimensions and the specifications of the end mill are shown in Figure 2 and Table 2.

### 3.2. Temperature Measurement

**Figure 3:**
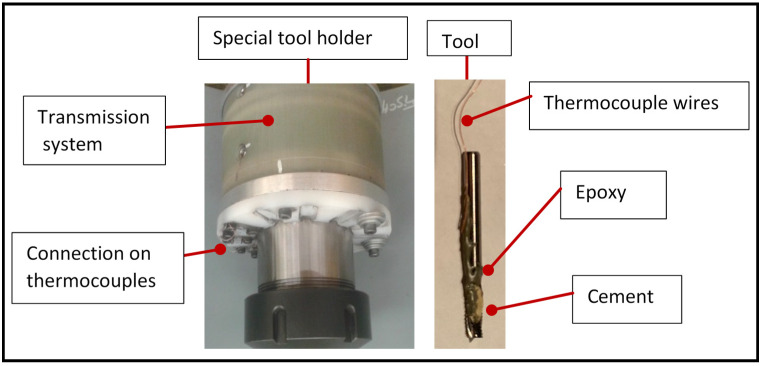
Schematic of cutting temperature measurement system. (Tool holder and tool with thermocouples).

Two K-type thermocouples (made from nickel–chromium wires each of 0.076 mm diameter (0.003 in.)) were used for measuring the temperature during machining. The characteristics of the thermocouples are presented in Table 3. Figure 3 shows a schematic representation of the cutting temperature/forces measurement system used. The thermocouples were installed on the tool, 2.2 mm from the tool tip to ensure the performance of temperature measurement. The thermocouple was held first by adhesive, then glued to the tooth by cement (OMEGABOND^®^ 400 # OB-400), which is an excellent thermal conductor. The tool was then left for 24 h in the open air. Once baked, the cement was covered with epoxy resin to protect it and ensure adhesion. The epoxy resin forms a sort of bridge between two teeth and protects the cement, which is very brittle, from the flow of chips. In all cases, the axial depth of cut was kept constant at 2 mm, such that the thermocouples were located at a distance of 0.2 mm from the cutting area. The tool was then mounted in a special holder (Type M-320, manufactured by Michigan Scientific Corporation, Charlevoix, MI, USA). It is equipped with connections for thermocouples and data were subsequently relayed and recorded (simultaneously with cutting force traces).

### 3.3. Dust Emission Measurement

Measurement of fine particles (diameter range 0.5–20 µm) was carried out using an Aerodynamic Particle Sizer (APS, model 3321, TSI Inc., Shoreview, MN, USA). Dust samples were sucked by a pump (1.5 L/min) through a suction tube, with the end of the tube placed near the machining area. The suction tube was connected to the dust measurement system, which consisted of the APS. The experimental scheme is illustrated in Figure 1.

### 3.4. Machining Parameters and Design of Experiments

Machining parameters such as cutting speed, axial depth of cut, and feed per tooth affect the cutting temperature. However, the cutting speed and feed per tooth are the more crucial and dominating factors. Therefore, the milling experiments emphasized on these two parameters, while the remaining parameters were set to be constants, e.g., the axial depth of cut was set as 2 mm. The two machining parameters that are studied are (a) feed per tooth (fz) and (b) cutting speed (vc). Three feed per tooth levels (2, 4, 6 micrometer/tooth), and three speed levels (10,000, 15,000, and 20,000 rpm) were examined in the experiments. To increase reliability and accuracy of the results, each test was repeated two times. A full factorial design of experiments across the two factors of feed, speed, and their corresponding levels requires a total of 3^2^ = 9 experiments. The cutting parameters and their levels are listed in Table 4.

## 4. Results and Discussions

### 4.1. Cutting Force

**Figure 4:**
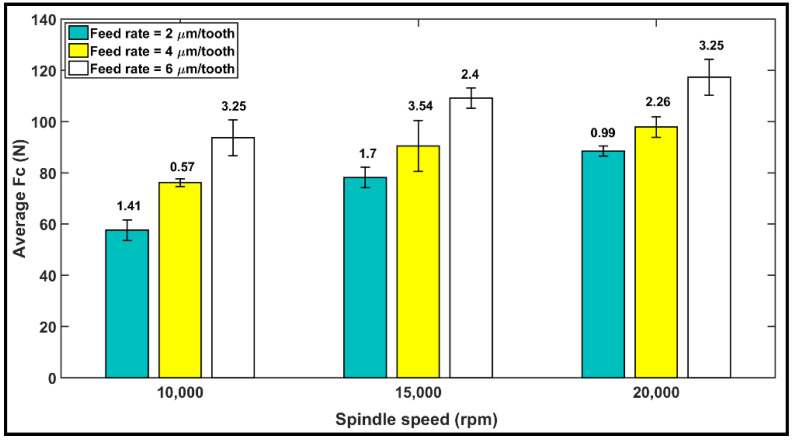
Variation of the average cutting forces with standard deviation at cutting speeds of 10,000, 15,000, and 20,000 rpm, and feed rates of 2, 4, and 6 µm/tooth.

The bar diagram in Figure 4 shows the average of the measured cutting forces Fc obtained from the cutting tests, with the error bars indicating standard deviation. Each bar diagram shows the magnitude of the average cutting forces during milling of the CFRP using a specific feed rate and spindle speed. From Figure 4, it can be seen that the cutting forces increase proportionally as the feed rate (i.e., chip thickness) increases. This phenomenon can be attributed to an increase in chip loads and volume of material removal at a higher feed rate, leading to an increase in the magnitude of Fc.

Our observation from Figure 4 is that under identical cutting conditions, cutting forces increase as the spindle speed increases from 10,000 to 20,000 rpm. The rate of the increase in cutting forces with cutting speed is believed to be associated with the cutting temperatures. Our observation of the tool cutting temperature is described in Section 4.2 and confirms the correlation between the increase in the cutting temperature and the cutting forces. This result may be explained based on an article by D. Wang et al. [49], which states that cutting speed has two opposing effects on the mechanics of chip formation. A higher cutting speed raises the chip’s strain rate, potentially resulting in increasing the cutting forces. At the same time, high-speed cutting generates more heat, which would soften the material and minimize the forces. In a previous study [50] on a CFRP composite using atomization-based cutting fluid sprays, it was reported that cutting forces decrease with an increase in cutting speed when machining a CFRP. In the experiments conducted in this work, however, we consistently observed higher forces at the higher (20,000 rpm) speed than at the lower (10,000 rpm) speed. Therefore, it may be concluded here that depending on the workpiece material, tool geometry, and cutting condition, either of these effects may dominate the process and increase or decrease the cutting forces.

### 4.2. Cutting Temperature

**Figure 5:**
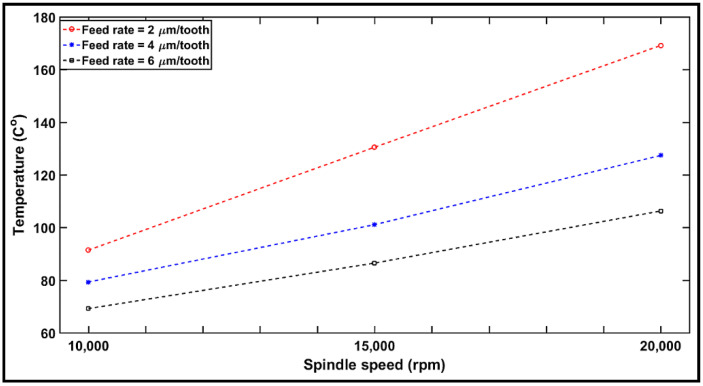
Variation in cutting temperature as a function of spindle speed and feed rate.

Figure 5 shows the evolution of the temperature registered during milling with respect to spindle speeds of 10,000, 15,000, and 20,000 rpm (i.e., vc = 100, 150, and 200 m/min) at the feed rates of 2, 4, and 6 µm/tooth, respectively, after machined distances Lc = 105 mm. The machining temperatures range from 69 to 170 °C. It can be also seen from Figure 5 that the overall machining temperature increases with an increase in spindle speed from 10,000 to 20,000 rpm. This is could be due to the increase in the friction between the cutting tool and the machined surface [27]. S. Gara et al. [51] measured the cutting temperature during machining of a multidirectional CFRP laminate. The experiments were conducted on a computer numerical control (CNC) machine with the cutting speed ranging from 80 to 200 m/min and the feed rate from 0.008 to 0.060 mm/tooth. The data were analyzed in order to establish empirical models showing the dependence of cutting temperature on tool geometry and cutting conditions. Based on the results, it was concluded that cutting speed is the factor influencing cutting temperature the most.

Considering the impact of feed rate, the cutting temperature decreased with an increase in the feed rate. This is in agreement with the common knowledge that, at high material removal rates (high feed per tooth), the largest share of heat is absorbed by the chip [19]. In the case of low material removal rates (low feed per tooth), the heat is absorbed equally by the chip and the tool. Further, with increasing material removal rates, the chip transports much more heat from the active area, whereas at low material removal rates, the portion of the heat conducted into the tool plays a more significant role [27]. This behavior is clearly observed in Section 4.4 upon analysis of the specific cutting energy.

As Figure 5 reveals, the machining temperatures recorded are still far from the glass transition temperature of the tested CFRP (*Tg* = 187 °C). In this case, it can be assumed that there is no thermal damage. The present study found that the CFRP machined using a spindle speed of 10,000 rpm (i.e., vc = 100 m/min) and 6 µm/tooth feed rate exhibited a reduced cutting temperature during milling under dry conditions. Based on the above analysis, it may be concluded that reduced chip thickness (lower feed rate/higher spindle speed) generates the highest temperatures, resulting in increased thermal damage. In contrast, increased chip thickness (higher feed rate/lower spindle speed) generates the lowest temperatures.

### 4.3. Validation of Modeling Temperature Results

**Figure 6:**
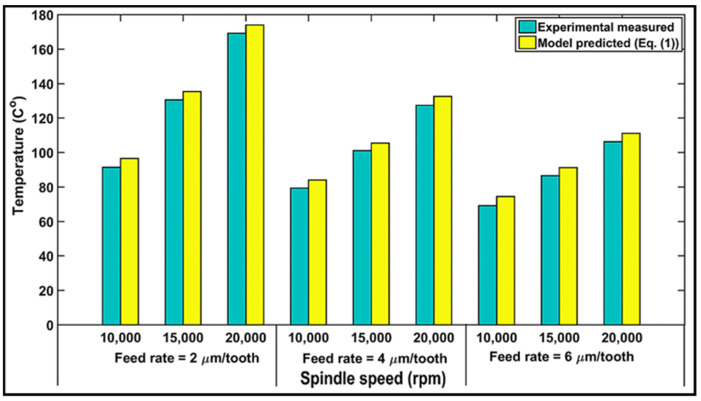
Comparison of measured and predicted temperatures as a function of spindle speeds and feed rates.

For validation purposes, a comparison between the cutting temperature measured by the K-type thermocouple from the experimental tests and the predicted cutting temperature calculated based on the proposed model, Equation (1), under the same cutting conditions, is performed. The results are displayed in Figure 6. It should be noted that the cutting conditions for the milling experiments were presented in Section 3.4, while the model was performed for material parameters of the CFRP cited from Ref. [41] and given in Table 1. Figure 6 also illustrates the maximum temperature generated during milling with respect to feed rate at spindle speeds of 10,000, 15,000, and 20,000 rpm (i.e., 100, 150, and 200 m/min). The simulated value of the temperature with respect to speed is found to rise with an increase in spindle speed and is predicted to be minimum for the 10,000-rpm speed (i.e., vc = 100 m/min). This agrees with the experimental results. A good agreement was also observed between the experimental and predicted values of the temperature for the feed rate, similar to Figure 6. The relative errors between the predicted and experimental values of temperature are found to be about 7%. Nevertheless, because the temperatures were measured at 0.2 mm from the axial depth of cut, *ap*, it could be argued that the temperature is higher for the model than for the experiments. Consequently, it may be summarized that the model can predict the temperature with a good degree of accuracy.

### 4.4. Specific Cutting Energy

The experimental cutting forces data were used to estimate the energy involved in the milling process (see Equation (4)).

**Figure 7:**
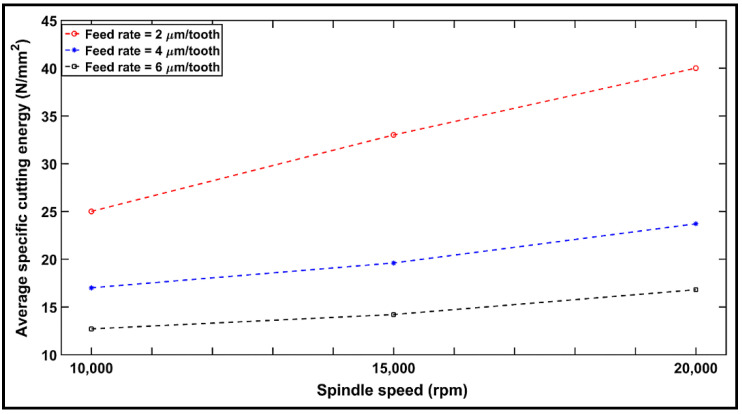
Variation of average specific cutting energy (Equation (4)) as a function of spindle speeds and feed rates.

Specific cutting energy (or specific cutting pressure), defined as the cutting force per unit area of the uncut chip, was calculated based on Equation (4), as shown in Figure 7. The chip thicknesses *ac* of each laminate ranged from 0.0023 to 0.0044 mm, corresponding to the feed per tooth values of 2, 4, and 6 µm/tooth. It can be seen in Figure 7 that the average specific cutting energy for the cutting force Fc decreased with an increase in feed rate but increased when the spindle speed increased. This is due to the fact that specific cutting energy is critically dependent on the feed rate (i.e., chip thickness), which is a function of the cutting engagement angle, *φ*. Our observation of the specific cutting energy is described in Section 2, Equation (6), which confirms the correlation between the increase in the specific cutting energy and the feed rate (i.e., chip thickness). It also indicates that the highest specific cutting energy recorded is 40.7 N/mm^2^ at 20,000 rpm (vc = 200 m/min) and a feed rate of 2 µm/tooth (0.002 mm/rev) and the lowest specific cutting energy is 12.7 N/mm^2^ at 10,000 rpm (vc = 100 m/min) and a feed rate of 6 µm/tooth (0.006 mm/rev). However, the specific cutting energy for CFRP composites is well below that of metals. The comportment of specific cutting energy regarding cutting speed is similar to that of the cutting force (Section 4.1), whereas it varies considerably with feed rate. A significantly higher specific cutting energy is required for removing small chips (small feed rate). For a high cutting speed and large chip size, the specific cutting energy for the tested composite tends to become constant. Therefore, from a material removal rate point of view, better machinability of CFRPs can be achieved with high feed rates and cutting speeds.

### 4.5. Study of Tool Wear

**Figure 8:**
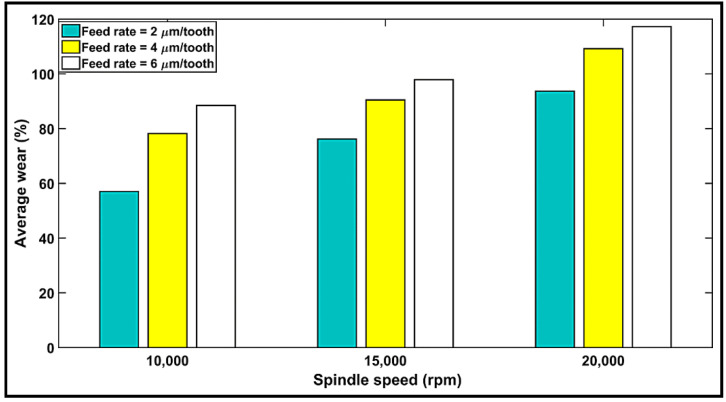
Variation in tool wear (%) with respect to spindle speeds and feed rates after cutting a distance Lc of 105 mm.

Dry machining of CFRP has an adverse impact on the operator as well as the environment in general. Therefore, a detailed study was undertaken to investigate tool wear under dry conditions using a router end mill. Tool wear was measured quantitatively based on the tool wear weight method, which is defined as the difference in tool weight before the machining and tool weight after the machining and is usually expressed as a percentage. Figure 8 illustrates the progression of tool wear (%) vs. the cutting conditions for a cutting distance of Lc = 105 mm. It can be observed that the tool wear increases when the feed rate (i.e., chip thickness) is increased. This can be directly related to the increase in the forces required to remove the larger chip thickness. This result also explains the high cutting force observed at 6µm/tooth feed rate in Section 4.1. Moreover, an increasing spindle speed offers a wider contact surface between the tool and the workpiece, thus resulting in superior surface contact and greater time consumption. As a result, an increase in tool wear is observed [39]. One can also conclude from Figure 8 that a significant improvement in the machinability of a CFRP in terms of tool wear can be achieved by decreasing the feed rate and spindle speed.

### 4.6. Particle Emission during Milling

**Figure 9:**
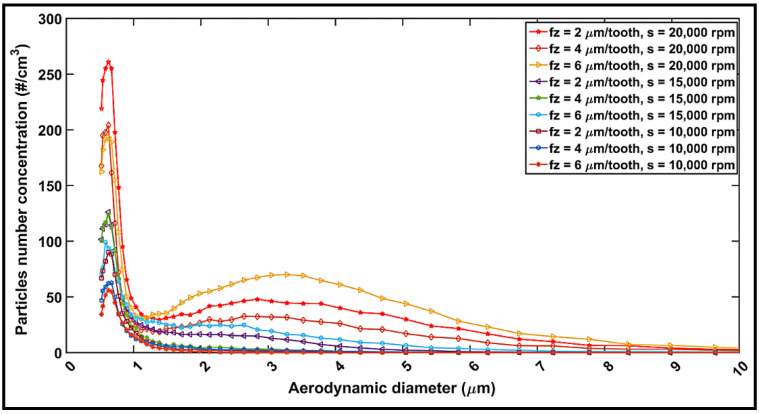
Number of fine particles vs. particle size during milling of CFRP for different cutting conditions.

**Figure 10:**
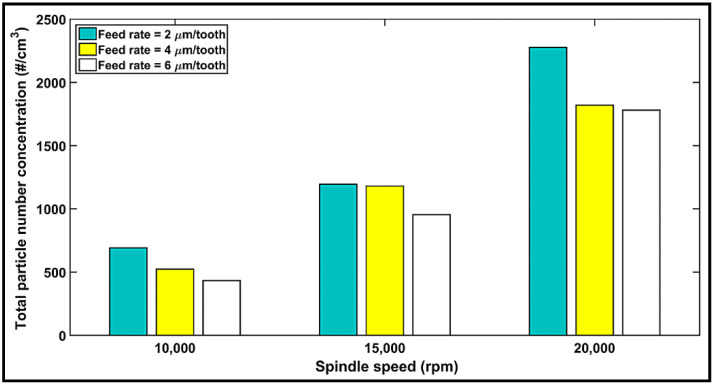
Total particles number concentration related to particle size and cutting parameters.

The maximum number of dust particles corresponding to different sizes as obtained in the experiments performed are plotted in Figure 9, depicting the peak-to-peak magnitudes for all cutting conditions. According to the collected data, the highest numbers of particles were registered for particles in the range 0.5–1 μm, followed by those with sizes between 2 and 7.5 μm, indicating a decrease in the number of particles with an increase in the feed rate, but an opposite effect with an increase in the spindle speed. Overall, the highest numbers of particles are obtained at 20,000 rpm spindle speed with 2 µm/tooth feed rate for 0.5–1 µm sized particles, as is also the case for the 1–7.5 µm sized particles. In contrast, at a 10,000-rpm spindle speed with a 6 µm/tooth feed rate, minimum numbers of particles are obtained. The following two important points were consistently noted in all the experiments: firstly, regardless of the feed rate used or the highest number of particles, the size of particles increased with speed, ranging from 0.5 to 2.5 µm at 10,000 rpm, and 0.5 to 7.5 µm at 20,000 rpm. This is explained by the higher cutting temperatures, as elaborated in Section 4.2 where an increase in the temperature was correlated with an increase in the number of particles. The second observation was that regardless of the spindle speed or the peak emission, the 2 and 4 µm/tooth feed rates displayed similar highest number of particles at a 15,000-rpm speed. However, when using a feed rate of 2 µm/tooth, a second peak of particle emission appears for particle size ranging from 2.0 to 2.5 µm. Likewise, the 4 and 6 µm/tooth feed rates offered the same highest number of particles at a 20,000-rpm speed. This observation is evident from the total particle number concentration (see Figure 10). However, the feed rate seems to have a limited influence as compared to that of speed. This is also observable in the analysis of variance (ANOVA) presented in Table 5. The influences of spindle speed and feed rate can be explained by the chip formation process during the machining of composites [38,52]. The shearing process, the chip separation and deformation, and the frictions in the shearing zone and at the chip–tool and tool–workpiece interfaces produce a lot of dust [53]. At low speeds, the chip crack is controlled by its brittleness. Therefore, there is limited contact and friction between its lips due to the crack opening [54]. A burr tool would generate a minimum number of particles when compared to a flat (traditional) tool, as reported by Haddad et al. [39]. Thus, it can be concluded that tool geometry is an important factor in dust emission. One can also conclude from Figure 9 and Figure 10 that in order to reduce the dust emission at low spindle speed, chip thickness should be increased by either decreasing the spindle speed or increasing the feed rate.

### 4.7. Analysis of Variance (ANOVA)

The main purpose of ANOVA is to use a statistical method to appreciate the effects of individual controlling parameters on the results obtained. The analysis was carried out at a 5% significance level (i.e., 95% confidence level). The significance of control factors in ANOVA is determined by comparing the F values of each control factor, as described in Table 5. The factors that produced statistically significant effects were selected to develop the experimental prediction model.

According to (a) in Table 5, in the analysis of variance for the cutting force, for example, two factors, namely feed rate and spindle speed, have *p*-values below 0.05, which makes them statistically significant at the 95% confidence interval. The speed has the greatest effect on the cutting force, followed by the feed. These factors were used to develop the empirical model. It is also seen that the interactions f * f, s * s, and f * s are not statistically significant for the cutting force, which was, therefore, not selected to develop the empirical model. Similarly, calculations were applied for other factors in evaluating their significance, as shown in (b–e) in Table 5.

(b) in Table 5 presents the analysis of variance for specific cutting energy, with two of the factors displaying *p*-values less than 0.05, indicating their statistical significance at the 95% confidence level. The feed rate had the highest effect on the specific cutting energy.

With respect to temperature, (c) in Table 5 indicates that the speed was statistically significant followed by the feed rate and interaction f * s, while the interactions f * f and s * s had no influence.

It can be seen from (d) in Table 5 that the feed rate had the greatest effect on tool wear, with the spindle speed as the next most influent factor. The fine particle emission was also analyzed. In this work, the fine particles studied have diameters ranging from 0.5 to 10 mm.

According to (e) in Table 5, the spindle speed (F-Ratio = 428.28) with a *p*-value of 0.000 is most significant, while the feed rate and interaction s * s are less significant at the 95% confidence level. The observations of the fine particle emission as described in Section 4.6 confirm the relationship between the increase in the total number of particles emitted and spindle speed.

**Figure 11:**
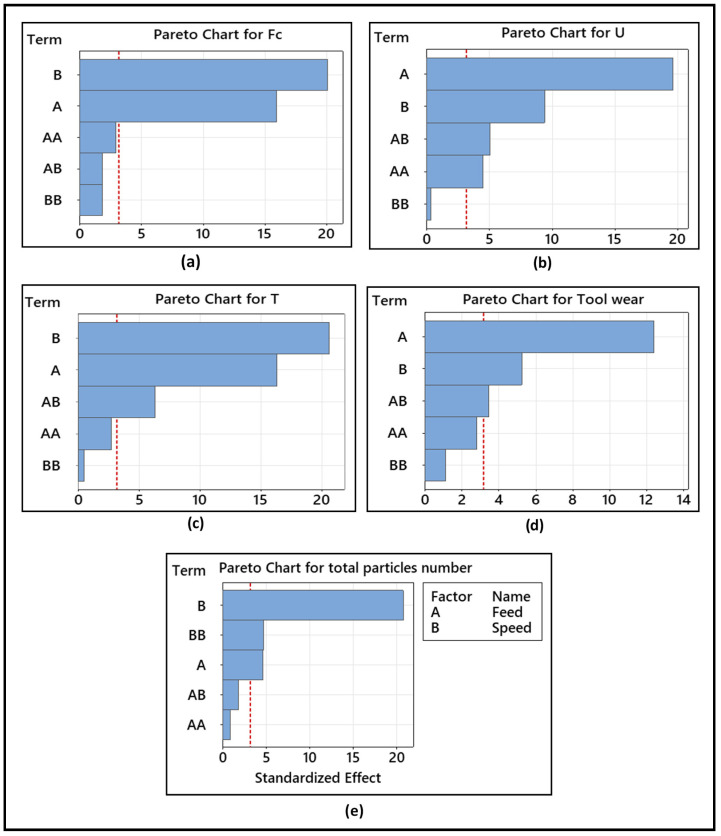
Pareto charts of the standardized effects of (**a**) cutting force Fc, (**b**) specific cutting energy U, (**c**) temperature T, (**d**) tool wear, and (**e**) total particle numbers concentration. A (feed rate), B (speed) AB interaction between feed rate (A) and speed (B).

The Pareto diagrams in Figure 11 illustrate the effect of the input parameters (feed and speed) on the output parameters (cutting force, temperature, specific cutting energy, tool wear, and dust emission). It can be seen from Figure 11 that the feed rate has a significant influence on specific cutting energy and tool wear, while spindle speed was shown to be the most effective factor with respect to the cutting force, temperature, and total particles number.

#### 4.7.1. Response Surface Methodology

The response surface methodology (RSM) establishes a mathematical relationship between two sets of data [55] with one set being the independent variables, i.e., cutting speed and feed rate, whereas the other set is the dependent variables or quality characteristics, i.e., cutting force, specific cutting energy, temperature, tool wear, and dust emission. This mathematical relation can be either first order (linear) or second order (quadratic). In the present work, RSM models were applied using a statistical software Minitab® 20.3 to establish relationships between the input parameters (independent variables) and the output responses (dependent variables). R-squared is a statistical measure of how close the data are to the fitted regression line. Another correlation coefficient often used is the adjusted R-squared, which is a modified version of R^2^ that has been adjusted for the number of predictors in the model. While the adjusted R^2^ also indicates how well terms fit a curve or line, it adjusts for the number of terms in a model and helps in determining if a new term improves a model. The correlation coefficients R^2^ and R^2^ adjusted for output parameters (cutting force, specific cutting energy, temperature, and total particle numbers) are given in Table 6. The results obtained for the coefficients indicate that the model used is quite adequate for prediction purposes. The predictive fit model equations for cutting force, specific cutting energy, temperature, tool wear, and total particle numbers were obtained as follows:

(9) $$F_{c}\left(N\right)=16.55+6.35f+\left(3.2\cdot 10^{-3}s\right)$$

(10) $$U\left(\frac{N}{{\mathrm{mm}}^{2}}\right)=22.62-7.63f+2.04\cdot 10^{-3}s+0.92\cdot f^{2}-\left(3\cdot 10^{-4}f\cdot s\right)$$

(11) $$T\left({}^{\circ}C\right)=7.3-4.5f+\left(9.5\cdot 10^{-3}s\right)-\left(1.02\cdot 10^{-3}f\cdot s\right)$$

(12) $$Tool\;wear\;\left(\%\right)=8.3-2.37f-\left(1.57\cdot 10^{-4}s\right)+0.3f^{2}+10^{-4}fs$$

(13) $$TPn\;\left(\frac{\neq }{{\mathrm{cm}}^{3}}\right)=1900-957f-0.23s+\left(1.36\cdot 10^{-4}s^{2}\right)$$

where *f* (μm) is the feed per tooth, *s* (rpm) is the spindle speed, and *TPn* the total particles number.

#### 4.7.2. Response Surfaces and Contour Plots for Output Parameters

**Figure 12:**
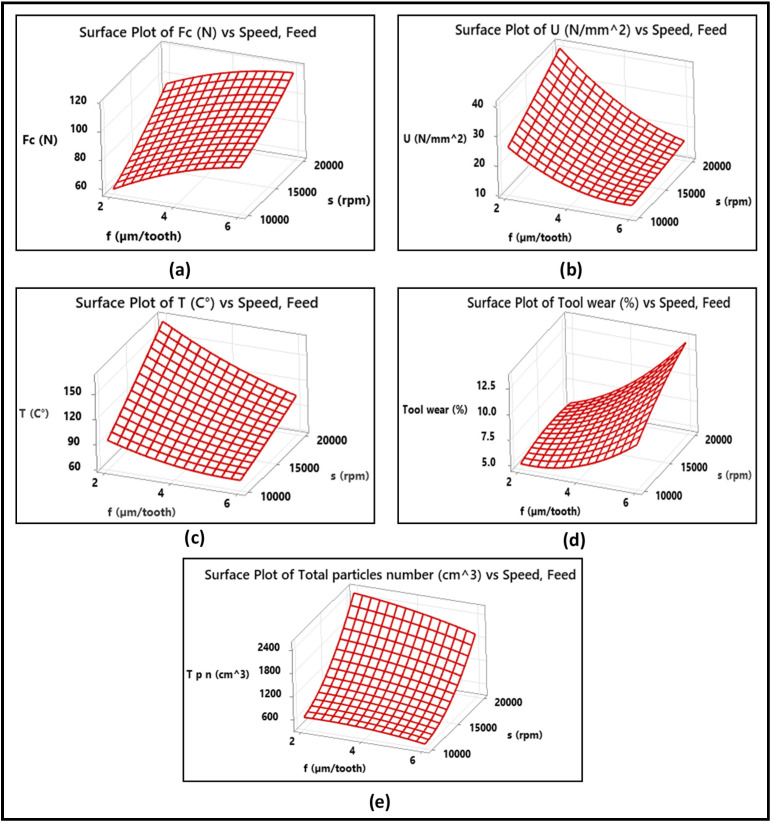
3D surface plots showing effect of cutting parameters on (**a**) cutting force, (**b**) specific cutting energy, (**c**) temperature, (**d**) tool wear, and (**e**) total particles number.

**Figure 13:**
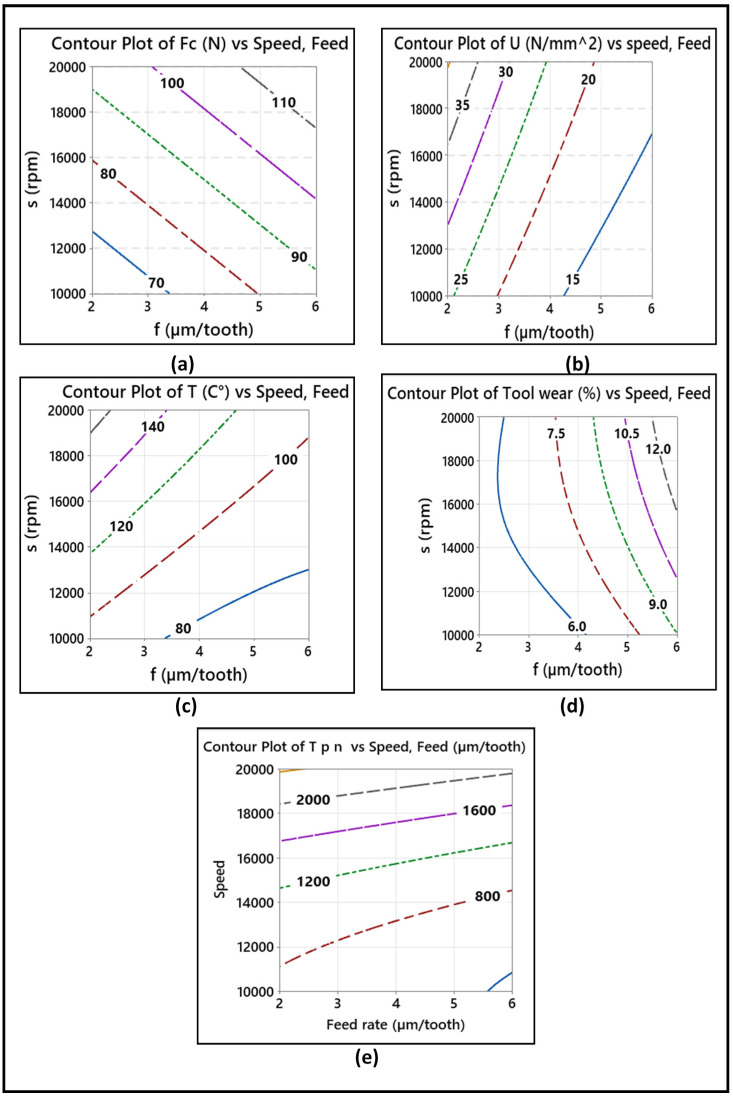
Corresponding contour plots showing effect of cutting parameters on (**a**) cutting force, (**b**) specific cutting energy, (**c**) temperature, (**d**) tool wear, (**e**) total particles number Tpn (≠/cm^3^).

Three-dimensional (3D) response surfaces and the corresponding two-dimensional (2D) contour plots were determined for the modeled parameters as a function of the independent factors, feed per tooth, and spindle speed. The response surfaces due to the effects of these two factors on the cutting force, specific cutting energy, temperature, tool wear, and dust emission are illustrated in Figure 12. The corresponding contour plots are shown in Figure 13. As presented by Figure 12a,d, the cutting force and tool wear increase for all three feed rates as the spindle speed increases from 10,000 to 20,000 rpm. The obtained maximum cutting force (Fc) and tool wear are 110 N and 12%, respectively, at a feed rate of 6 µm/tooth and a spindle speed of 20,000 rpm. On the other hand, from Figure 12b,c, the specific cutting energy and the temperature decrease significantly for all three feed rates and increase as the spindle speed increases from 10,000 to 20,000 rpm. The obtained maximum specific cutting energy (U) and temperature (°C) are 35 N/mm^2^ and 140 °C, respectively, at a feed rate of 2 µm/tooth and a spindle speed of 10,000. Figure 12e shows that the total particles number increases significantly with the increase in spindle speed from 10,000 to 20,000 rpm and decreases as the feed rate increases. The obtained maximum total particles number is 20,000 cm^3^ at 20,000 rpm and a feed rate of 2 µm/tooth. In addition, the elliptical nature of the contour lines in Figure 13d,e implies that there is a significant interaction between the feed rate and the spindle speed. The interactions between the independent variables in the contour plots have an important impact on the response because high interactions mean the existence of maximum, minimum, or saddle points in the response surface, which help in estimating the optimization process. On the other hand, in the contour plots in Figure 13a–c, the nearly linear contour lines imply that interaction between the feed rate and the spindle speed in the case of the corresponding output parameters is weak.

#### 4.7.3. Analysis of Responses

**Figure 14:**
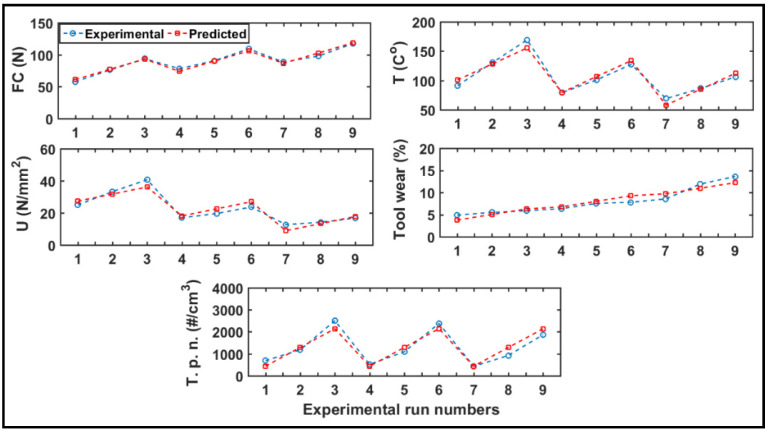
Comparison between experimental data and predicted data.

The values from the experiment and the predicted values obtained from the empirical models (Equations (9)–(13)) are shown in Figure 14. Table 7 compares the values of the optimum cutting parameters for output parameters and their predicted values (the optimal spindle speed was 10,000 rpm). It is concluded that a low cutting speed and a low feed rate are preferred to minimize the cutting force and the tool wear. In addition, in order to maintain a low temperature, specific cutting energy, and dust emission levels, a low cutting speed and a high feed rate are preferred. Such a combination would help in maintaining an acceptable removal rate and thus render good productivity.

## 5. Conclusions

The present study attempted to investigate routing milling of CFRPs under dry machining conditions. The effect of cutting parameters (feed rate, spindle speed) on the cutting force, cutting temperature, tool wear, and dust emission was assessed. From the experimental results and statistical analysis of the data, the following may be concluded.

The feed rate has a greater effect on cutting force and tool wear than the spindle speed in the milling of CFRP composite materials but has no influence on dust emission.

1. Cutting speed is the main parameter that controls the cutting temperature in milling of CFRP composite materials, followed by feed rate.
2. The predicted temperatures from the analytical model agreed well with the experimental observations within a range of ±10%.
3. The cutting temperature does not exceed the glass transition temperature for the cutting speeds (10,000, 15,000, 20,000 rpm) and feed rates (2, 4, 6 µm/tooth) used.
4. The specific cutting energy for the cutting forces considered was investigated as a material property. It was found to increase with an increase in the spindle speed but decrease with an increase in the feed rate.
5. During the machining, fine particles were emitted (aerodynamic diameters ranging from 0.5–10 µm). The maximum concentration of fine particles reached 2776.6 #/cm^3^, while the minimum number reached 432.3 #/cm^3^. The spindle speed significantly affects fine dust generation, whereas the feed rate is not statistically significant. The total number concentration of fine particles decreased with an increase in the feed rate.
6. The optimum levels of the control factors for minimizing the cutting force, tool wear, cutting temperature, specific cutting energy, and fine particles emission were derived using the ANOVA approach. The optimal conditions for cutting force and tool wear were observed at cutting speed = 10,000 rpm and feed rate = 2 µm/tooth, while those for a specific cutting energy, cutting temperature, and total number of particles were observed at cutting speed = 10,000 and feed rate = 6 µm/tooth.

## Figures and Tables

**Table 1:** Mechanical and physical properties of CFRP used.

Property	Value
Volume of fibers in each tape	54%
Young’s modulus of the fibers	225 GPa
Young’ modulus of the sheets	65 GPa
Density (ρ)	1.81 g/cm^3^
Heat capacity (c)	0.06 J/g·°C
Thermal conductivity (α)	0.5 j/s-mm °C

**Table 2:** Specifications of the tool used in the experiments.

Cut Diameter (mm)	Rake Angle (Degrees)	Flute Length (mm)	No of Flutes	Overall Length (mm)
3.175	6	6.477	6	38.1

**Table 3:** Characteristics of thermocouples.

Brand	OMEGA^®^	
Type	K	Reference
Red—Positive	Chrome–Nickel	CHROMEGA^®^ TFAL-0.003 (Ø 0.076 mm)
Yellow—Negative	Aluminum–Nickel	ALOMEGA^®^ TFCY-0.003 (Ø 0.076 mm)

**Table 4:** Machining parameters and selected levels.

Factors	Level 1	Level 2	Level 3
s: spindle speed (rpm)	10,000	15,000	20,000
fz: feed per tooth (µm)	2	4	6
Lc: cutting length (mm)	105		
*ap*: axial depth of cut (mm)	2		

**Table 5:** Analysis of variance (ANOVA) for output factors.

**(a)** **ANOVA for Cutting Force Fc**					
Source	DF	SS	MS	F-Ratio	*p*-Value
f	1	967.74	967.74	254.87	0.001 *
s	1	1532.80	1532.80	403.69	0.000 **
f * f	1	33.62	33.62	8.85	0.059
s * s	1	13.00	13.00	3.43	0.161
f * s	1	13.32	13.32	3.51	0.158
Error	3	11.39	3.80	-	
Total	8	2571.88	-		
**(b)** **ANOVA for Specific Cutting Energy U**					
f	1	509.682	509.682	385.69	0.000 **
s	1	117.042	117.042	88.57	0.003 *
f * f	1	27.134	27.134	20.53	0.020
s * s	1	0.161	0.161	0.12	0.750
f * s	1	33.640	33.640	25.46	0.015
Error	3	3.964	1.321	-	
Total	8	691.622	-		
**(c)** **ANOVA for Temperature T**					
f	1	2777.80	2777.80	267.40	0.000 **
s	1	4428.17	4428.17	426.27	0.000 **
f * f	1	78.13	78.13	7.52	0.071
s * s	1	2.42	2.42	0.23	0.662
f * s	1	414.12	414.12	39.87	0.008 *
Error	3	31.16	10.39	-	
Total	8	7731.80	-		
**(d)** **ANOVA for Tool Wear**					
f	1	52.8185	82.8185	153.80	0.001 **
s	1	9.4627	9.4627	27.55	0.013 *
f * f	1	2.7036	2.7036	7.87	0.068
s * s	1	0.4377	0.4377	1.27	0.341
f * s	1	4.1657	4.1657	12.31	0.040
Error	3	1.0303	0.3434	-	
Total	8	70.6185	-		
**(e)** **ANOVA for Fine Particle Emission**					
f	1	219,984	219,984	21.68	0.019 *
s	1	4,345,614	4,345,614	428.28	0.000 **
f * f	1	8577	8577	0.85	0.426
s * s	1	222,814	222,814	21.96	0.018 *
f * s	1	31,134	35,134	3.46	0.160
Error	3	30,440	10,147	-	
Total	8	4,862,563	-		

**: most influent factor; * Second most influent factor.

**Table 6:** Correlation coefficients R^2^ and R^2^ adjusted for Equations (9)–(13).

Equation No.	Output Parameters	R^2^	R^2^ Adjusted
1	Cutting force	97.2%	96.3%
2	Specific cutting energy	99.4%	98.8%
3	Temperature	98.5%	97.7%
4	Tool wear	97.9%	95.8%
5	Total particle numbers (Tpn)	98.4%	97.5%

**Table 7:** Comparison between experimental data and predicted data for the recommended spindle speed.

No.	s (rpm)	f (µm/Tooth)	Responses		
			Variables	Exp.	Predicted
1		2	Force (N)	57.6	61.2
2		6	U (N/mm^2^)	12.7	8.92
3		6	T (°C)	69.20	58.08
4		2	Tool wear	4.89	3.78
5		6	Total number of particles (#/cm^3^)	432.3	438.03

## Data Availability

Not applicable.

## Nomenclature

CFRP	Carbon fiber reinforced polymer
*Fx*	Normal force in the x direction
*Fy*	Feed force in the y direction
*Fc*	Cutting force (N)
∅	Engagement angle (°)
*α*	Rake angle (°)
U	Specific energy (Nm/mm^3^)
vc	Cutting speed (m/min)
s	Spindle speed (rpm)
SS	Total sum of squares
ANOVA	Analysis of variance
CNC	Computer numerical control
R^2^-adj.	The percentage of variation explained by only the independent variables that actually affect the dependent variable.
ρ	Density (g/cm^3^)
C	Heat capacity (J/g-C)
K	Thermal diffusivity of work material (mm^2^/s)
T	Temperature rises at tool-chip interface (°C)
*α*	Thermal conductivity (j/s-mm °C)
Cρ	Specific heat (j/mm^3^-C)
MRR	Material removal rate (N-m/mm^3^)
ap	Depth of cut (mm)
DF	Degrees of freedom
MS	Mean of squares
µ	Micrometer
R^2^	The correlation between the predicted values and the observed values
